# Supplementary material for: Metabolomics in Pulmonary Hypertension—A Useful Tool to Provide Insights into the Dark Side of a Tricky Pathology
Source: Int J Mol Sci. 2023 Aug 25;24(17):13227. doi: 10.3390/ijms241713227 (PMC10487467; doi:10.3390/ijms241713227)
Supplement: Supplementary file 1 [file ijms-24-13227-s001.zip › ijms-2523844-supplementary.pdf]

# PRISMA Flow Diagram

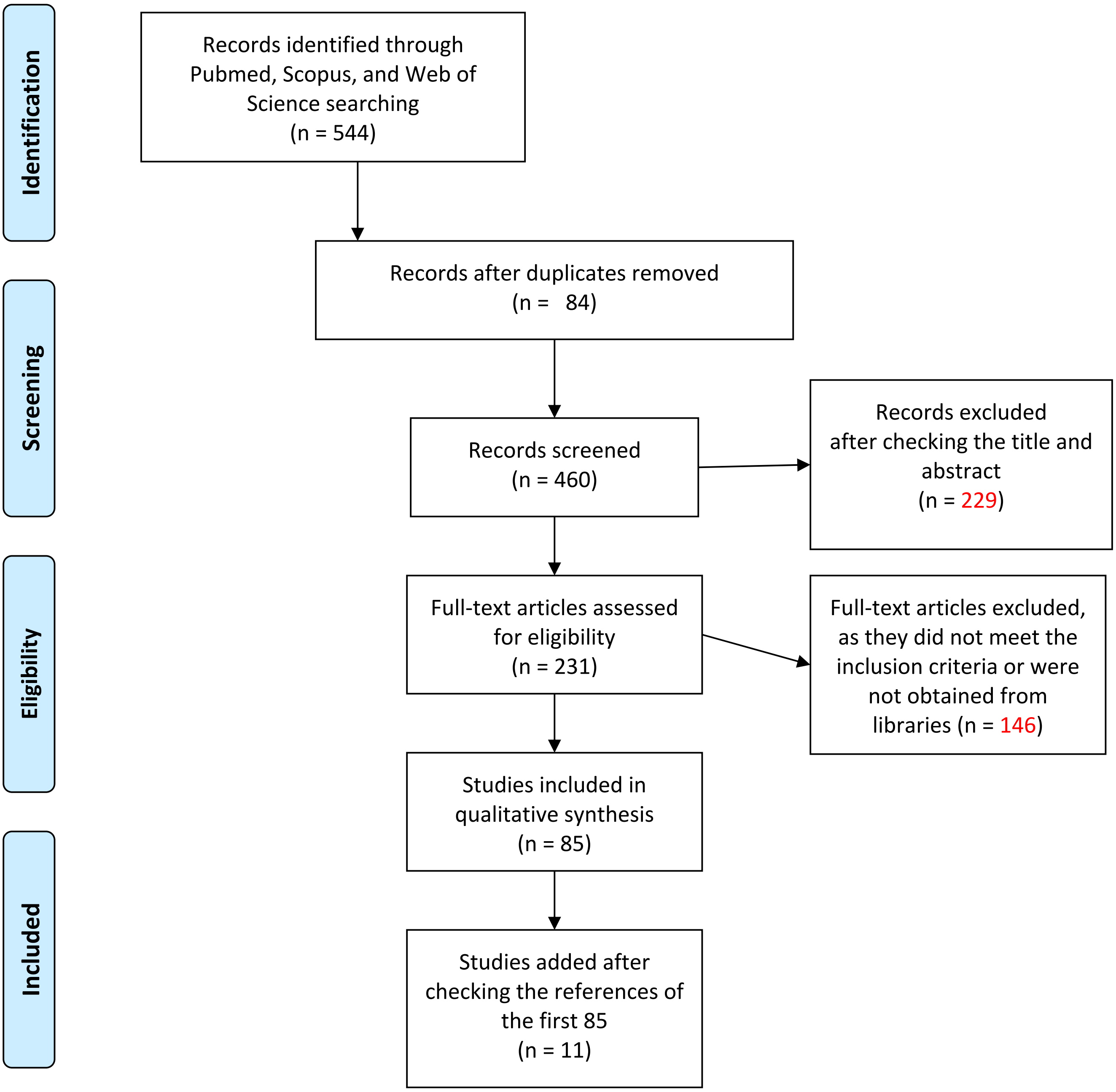


*From:* Moher D, Liberati A, Tetzlaff J, Altman DG, The PRISMA Group (2009). *P*referred *R*eporting *I*tems for *S*ystematic Reviews and *M*eta-*A*nalyses: The PRISMA Statement. PLoS Med 6(6): e1000097. doi:10.1371/journal.pmed1000097

**For more information, visit www.prisma-statement.org.**
